# Oral health-related quality of life in Egyptian children with Molar Incisor Hypomineralisation. An observational study

**DOI:** 10.1038/s41405-024-00272-x

**Published:** 2024-11-30

**Authors:** Lamia Khairy Gadallah, Eman Korayem, Reem Wahby

**Affiliations:** 1 Researcher, Orthodontics and Pediatric Dentistry Department, National Research Centre, Giza, Egypt; 2 Lecturer in Pediatric Dentistry and Dental Public Health Department, Faculty of Dentistry, Cairo University, Giza, Egypt

**Keywords:** Oral diseases, Diseases

## Abstract

**Aim:**

The aim of this study was to study the effect of Molar Incisor Hypomineralisation (MIH) on Oral Health-Related Quality of Life (OHRQoL) in children at the mixed dentition stage and correlate it with their caries experience.

**Subjects and methods:**

One hundred two children aged from 8 to 10 years were recruited, 51 with MIH and 51 as control. Caries experience was recorded using DMFT/deft and ICDAS II. MIH-TNI index was used for classifying MIH severity. CPQ_8–10_ with its Arabic version was used to evaluate OHRQoL. Mann–Whitney *U* test and Kruskal–Wallis test were used for comparisons (*p* ≤ 0.05).

**Results:**

The mean value for CPQ_8–10_ in MIH group was 24.67 (±11.84; median 23; range 6–55) in comparison to 21.04 (±12.3; median 18; range 2–54) for the control group, with no significant difference between groups (*p* = 0.109). A significantly higher value (*p* = 0.011) was recorded in patients with MIH-TNI 4 mean 32.16 (±13.9; median 35; range from 7 to 55) in comparison to other categories of MIH.

**Conclusion:**

Children with and without MIH, with similar caries experience showed no difference in OHRQoL. However, the presence of the severe form of MIH including hypersensitivity and enamel disintegration negatively impacted those children’s OHRQoL.

## Introduction

Molar Incisor Hypomineralisation (MIH) is defined as “hypomineralisation of systemic origin of one to four permanent first molars frequently associated with affected incisors” [1]. This qualitative developmental defect of enamel is characterized by demarcated opacities, soft and porous enamel that could be easily broken down and chipped after eruption exposing the underlying dentin and also reported hypersensitivities related to some of these affected teeth [2]. This phenomenon was first introduced with this terminology in 2001 [3].

The etiology of MIH is believed to be due to some systemic and genetic factors [4]. The genetic predisposition of MIH was proposed as some studies showed certain variants in amelogenesis related genes in MIH children and greater concordance of MIH in monozygotic twins [5, 6]. The systemic etiological hypotheses were related to prenatal exposures as maternal medications during pregnancy or perinatal exposures as premature birth, cesarean delivery, and hypoxia or postnatal exposures as early childhood illnesses in the first four years of life as asthma, pneumonia, bronchitis, ear or urinary tract infections and also medications as antibiotics [7, 8].

The worldwide prevalence rates of MIH had shown a wide range that varied from 2.4% to 40.2% [9, 10]. A recent national study reported a prevalence rate of 14.2% [11], and this coincides with the recent global mean prevalence rates of 14.2% and 12.9% [4].

Oral health-related quality of life (OHRQoL) is “a multidimensional construct that reflects among other things people’s comfort when eating, sleeping, and engaging in social interaction; their self-esteem; and their satisfaction with respect to their oral health” [12]. The quality of life of children with oral diseases, who are undergoing physical, mental, and social growth could be negatively affected, in contrast to children who do not have any oral pathology [13].

Children affected by MIH are more susceptible to caries affection and progression that can lead to pulpal inflammation [14]. Moreover, children tend to avoid tooth brushing because of increased sensitivity [1]. This hypersensitivity is an another major symptom that is related to many MIH affected teeth [15]. The hypersensitivity varies in severity from a mild response to an external stimulus to spontaneous hypersensitivity, with reported difficulty in achieving profound analgesia during restorative procedures [16]. Such factors can affect the daily lives of children, leading to negative social and esthetic effects as well as pain [17, 18], which in return negatively impact both the children’s oral self-perception [19], and the perception of their families towards their OHRQoL [20].

The European Academy of Pediatric Dentistry (EAPD) suggests MIH should be assessed in cross-sectional studies in 8-year-old children [17]. MIH presents a notable range of clinical manifestations that are subject to age-related influences [21]. Consequently, the assessment of MIH’s effect on OHRQoL in younger individuals is warranted, as it allows for the early detection of perceived needs in the disease’s initial phases. The Child Perceptions Questionnaire (CPQ_8–10_) is commonly utilized to assess OHRQoL in children aged 8–10 years, it is a generic questionnaire designed to cover different oral conditions including caries, malocclusion and craniofacial anomalies [22, 23].

Although MIH has been known for more than two decades now but no prior studies have examined MIH and OHRQoL in Egypt and to our knowledge there is lack of enough data about the relationship between them regionally in the Middle east, in addition to only one study has been previously reported in Africa [24].

It was also recommended in epidemiological studies on enamel hypominerlaization to use the International Caries Detection and Assessment System II (ICDAS II) for caries recording [25]. This is distinctively featured in this study, so that the relationship between MIH and caries is determined more accurately. The aim of this study is to investigate the effect of MIH on OHRQoL in children at their mixed dentition stage and correlate it with their caries experience.

## Methods

### Study design and setting

This was an analytical observational study of matched pairs. Patients were recruited between May 2023 and December 2023, from those who were seeking dental care at the Outpatient clinic of the Pediatric Dentistry Department in the Faculty of Dentistry and aged from 8 to 10 years.

Exclusion criteria included children with systemic diseases, any physical or mental disability, severe malocclusion, or any developmental defects other than MIH. Recruitment of patients continued until the total sample of 102 Egyptian children were collected as required where Group I consisted of 51 children suffering from MIH, and Group II included 51 children as controls without MIH.

Sample size calculation was performed using G power statistical power Analysis program (version 3.1.9.4) for sample size determination [26]. A total sample size *n* = 102; (subdivided into 51 in each group) was sufficient to detect a large effect size (d) = 0.69, with an actual power (1-β error) of 0.95 (95%) and a significance level (α error) 0.05 (5%) for two-sided hypothesis test, based on the results of a previous study by Velandia et al. [2], where MIH-affected children were significantly higher than control children in Total CPQ8-10 score showing MIH Median (interquartile range IQR) [12.5 (17)] versus [4 (3.5)].

### Clinical examination

All the participants were examined by two examiners. Before the clinical examination, the children were asked to brush their teeth under the examiner’s supervision. The children were examined on a dental chair. A plain dental mirror and the ball-ended explorer (WHO periodontal probe) were used with an air-water syringe and under artificial lighting of the dental unit. The examination proceeded in a clockwise direction starting from the upper right quadrant and ending by the lower right one.

### Dental caries detection methods

The caries experience of the children was recorded using two methods. The first one was DMFT [decayed (D), missing (M), and filled (F) permanent teeth] and deft [decayed (d), tooth indicated for extraction due to caries (e) and filled (f) primary teeth] caries indices [27, 28]. The second one was the ICDAS II caries index [29].

The examiners received training for caries recording and were calibrated. For ICDAS II recording, theoretical sessions and discussions for about six hours were held and the examiners were further trained using the ICDAS training online flashcards (https://quizlet.com).

Ten patients were examined firstly by the examiners and the results were checked with the senior and experienced examiner and inter-examiner reliability was statistically analyzed with Kappa coefficient [30].

### DMFT & deft recording indices

Caries experience was diagnosed through guidelines established by the World Health Organization (WHO) [31]. Assessment of dental caries in permanent teeth was done according to the DMFT index. Teeth with carious lesions that are frankly cavitated with detectably softened floor or wall or teeth with carious lesions adjacent to restorations were recorded as “D”. Any tooth with a temporary filling was also recorded as “D”. Missing teeth due to caries were recorded as “M”. Teeth with permanent fillings or with defective fillings but not decayed was counted as “F”.

Dental caries evaluation in primary teeth was done according to the deft index [27, 28].

### ICDAS II recording system

Following the ICDAS guidelines, all the teeth were examined wet first and then dried by the air-water syringe of the dental chair for 5 s each [30].

Each surface took a two-digit code where the first digit is for sealant and restoration and it ranges from 0 to 9, while the second digit is for coronal caries recording and it ranges from 0 to 6, it records caries related to pit and fissure, smooth surface (mesial and distal), free smooth surface (buccal, lingual or proximal surfaces without adjacent) and caries associated with restorations and sealants CARS [29]. Taking into consideration that surfaces with developmental defects as demarcated opacities were recorded as sound [32].

### MIH diagnostic criteria

The diagnosis of MIH was according to the criteria proposed by the EAPD [17, 33]. This includes (a) Well-demarcated opacities greater than 1 mm, which can be white, yellow, or brown in color; (b) Post-eruptive enamel breakdown where there is deficiency and surface loss of enamel after eruption; (c) Atypical restorations that are not conforming to the typical caries picture, they are extending to the buccal or lingual smooth surfaces and at the margins of the restorations frequently an opacity can be noticed; (d) Extracted first permanent molars whether with opacities, breakdowns or atypical restorations in the other first permanent molars or in dentitions with low caries activity in combination with demarcated opacities on the incisors, and (e) Failure of eruption of a molar or an incisor at 8 years of age.

### The MIH treatment need index (MIH-TNI)

The MIH-TNI was recorded according to the Würzburg MIH concept [34]. This classification is founded upon the two most important clinical features: hypersensitivity and destruction (disintegration). The recording is by visual means using a mirror and with tactile means using a probe and on drying with an air syringe.

The whole dentition is divided into sextants, recording starts in a clockwise direction, starting from the maxillary right sextant (distal to/with 14/54) then the maxillary front (with 13–23/53–63) then the maxillary left sextant (distal to/with 24/54), then the mandibular left sextant (distally to/with 34/74), then the mandibular front sextant (with 33–43/73–83), and ending by the mandibular right sextant (distal to/with 44/84) using a sextant recording diagram.

The essential parameters of the MIH including opacity, enamel fractures, and hypersensitivity are taken into consideration. The index values are 0 for no MIH and from 1 to 4 for the presence of MIH with further grading as shown in Table 1.

**Table 1:** MIH-TNI index

Index	Definition
Index 0	No MIH, clinically free of MIH
Index 1	MIH without hypersensitivity, without defect
Index 2	MIH without hypersensitivity, with defect
2a	≤1/3 defect extension
2b	≥1/3 ≤2/3 defect extension
2c	≥2/3 defect extension or/and defect close to the pulp or extraction or atypical restoration
Index 3	MIH with hypersensitivity, without defect
Index 4	MIH with hypersensitivity, with defect
4a	≤1/3 defect extension
4b	≥1/3 ≤\2/3 defect extension
4c	≥2/3 defect extension or/and defect close to the pulp or extraction or atypical restoration

### Evaluation of OHRQoL

OHRQoL was measured using the validated Arabic version of CPQ_8–10_ [35]_._ The CPQ_8–10_ questionnaire comprises 25 items categorized into four distinct domains: oral symptoms, functional limitations, emotional well-being (five items each), and social well-being (ten items). The questions evaluate the frequency of occurrences throughout the last month. The ratings are evaluated using a five-point Likert scale that ranges from 0 to 4 for each item, with 0 meaning “never,” 1 representing “once or twice,” 2 indicating “sometimes,” 3 for “often,” and 4 denoting “every day or almost every day”. Hence, total scores range from 0 to 100. A total score of zero indicates the absence of any problem, the higher the score, the worse OHRQoL. The CPQ_8–10_ includes two items for child identification (sex and age) and includes two global questions with a four-point Likert scales ranging from 0 to 3, the first assesses the child’s oral health where 0 is for “very good,” while 3 is for “poor” and the second evaluates the extent that his oral or facial condition affects his overall well-being, where 0 means “not at all” to 3 that means “a lot” [35, 36].

### Statistical analysis

Data management and statistical analysis were conducted utilizing the Statistical Package for Social Sciences version 20. Summary statistics for numerical data included measures such as mean, standard deviation, confidence intervals, median and range. The normality of data was assessed through examination of data distribution using Kolmogorov–Smirnov and Shapiro–Wilk tests.

Based on the non-parametric distribution of most data, groups were compared using the Mann–Whitney *U* test, while the Kruskal–Wallis test was used for comparisons based on the MIH grade. All *p*-values are two-sided. *P*-values ≤ 0.05 were considered significant.

## Results

### Study population

A total of 102 subjects aged from 8 to 10 years were included in this study (51 per each group). The mean age in the MIH group was 8.94 ± 1.45 years in comparison to 8.8 ± 1.55 years in the control group, with no significant difference between the two groups (*p* = 0.602). The MIH group included 19 males (37.3%) and 32 females (62.7%) while the control group included 24 males (47.1%) and 27 females (52.9%); with no significant difference between groups (*p* = 0.316).

### Caries recordings

The caries recordings for both DMFT/deft and ICDAS II indices were presented in Table 2.

**Table 2:** Descriptive statistics and comparison between groups regarding Caries recordings by DMFT/deft and ICDAS II indices (Mann–Whitney *U* test):

		Mean	Std. dev	Median	95% Confidence interval for mean		Min	Max	*P* value
					Lower bound	Upper bound			
D	MIH	1.33	1.34	1.00	0.96	1.71	0.00	4.00	0.001*
	Control	0.53	0.82	0.00	0.30	0.77	0.00	3.00	
M	MIH	0.00	0.00	0.00	0.00	0.00	0.00	0.00	0.155 ns
	Control	0.04	0.20	0.00	−0.02	0.09	0.00	1.00	
F	MIH	0.22	0.58	0.00	0.05	0.38	0.00	3.00	0.044*
	Control	0.04	0.20	0.00	−0.02	0.09	0.00	1.00	
DMFT score	MIH	1.55	1.39	1.00	1.16	1.94	0.00	4.00	0.000*
	Control	0.59	0.94	0.00	0.32	0.85	0.00	4.00	
d	MIH	2.24	2.16	3.00	1.63	2.84	0.00	8.00	0.055 ns
	Control	2.69	2.80	3.00	1.51	3.08	0.00	9.00	
e	MIH	0.39	0.70	0.00	0.20	0.59	0.00	3.00	0.448 ns
	Control	0.47	0.70	0.00	0.27	0.67	0.00	3.00	
f	MIH	0.59	1.36	0.00	0.21	0.97	0.00	6.00	0.029*
	Control	0.16	0.64	0.00	−0.02	0.34	0.00	4.00	
Deft score	MIH	3.72	2.57	3.00	3.09	4.14	0.00	12.00	0.245 ns
	Control	3.65	3.03	3.00	2.97	3.88	0.00	11.00	
DMFT + deft	MIH	4.76	2.84	5.00	3.97	5.56	0.00	13.00	0.452 ns
	Control	4.51	3.12	4.00	3.63	5.39	0.00	13.00	
ICDAS II (1–6)	MIH	9.16	3.86	9.00	8.07	10.24	3.00	20.00	0.205 ns
	Control	8.22	3.59	8.00	7.21	9.23	1.00	18.00	
ICDAS II (4–6)	MIH	4.41	2.62	4.00	3.68	5.15	0.00	12.00	0.469 ns
	Control	4.24	3.16	3.00	3.35	5.12	0.00	14.00	

Significance level *p* ≤ 0.05, *significant, *ns* non-significant

The DMFT score for caries index of permanent teeth was significantly (*p* = 0.000) higher in MIH group (mean 1.55 ± 1.39, median 1, range 0–4) in comparison to control group (mean 0.59 ± 0.94, median 0, range 0–4). The D score was significantly (*p* = 0.001) higher in MIH group in comparison to the control group, and the F score was significantly (*p* = 0.044) higher in the MIH group) in comparison to control group while there was no significant difference in M score (*p* = 0.155). However, there was no significant difference in deft score for primary teeth between MIH and control groups (*p* = 0.245).

The DMFT + deft score recorded mean 4.76 (±2.84, median 5, range 0–13) in MIH group, in comparison to mean 4.51 (±3.12, median 4, range 0–13) in control group. This difference was not statistically significant (*p* = 0.452).

The analysis for ICDAS II data was done at two levels concerning the second digit of coronal caries recording, the first level is that score 0 is for sound surfaces and scores (1–6) is for caries [37], the other level is that only scores (4–6) is for caries, and considered for evaluation as those scores are equivalent to the WHO definition of caries [30]. Each tooth received the worst second digit code for one of its five surfaces for caries recording [37].

ICDAS II (1–6) scores for both non cavitated and cavitated lesions recorded mean 9.16 (±3.86, median 9, range 3–20) in the MIH group, in comparison to mean 8.22 (±3.59, median 8, range 1–18) in the control group. This difference was not statistically significant (*p* = 0.205).

**Fig. 1:**
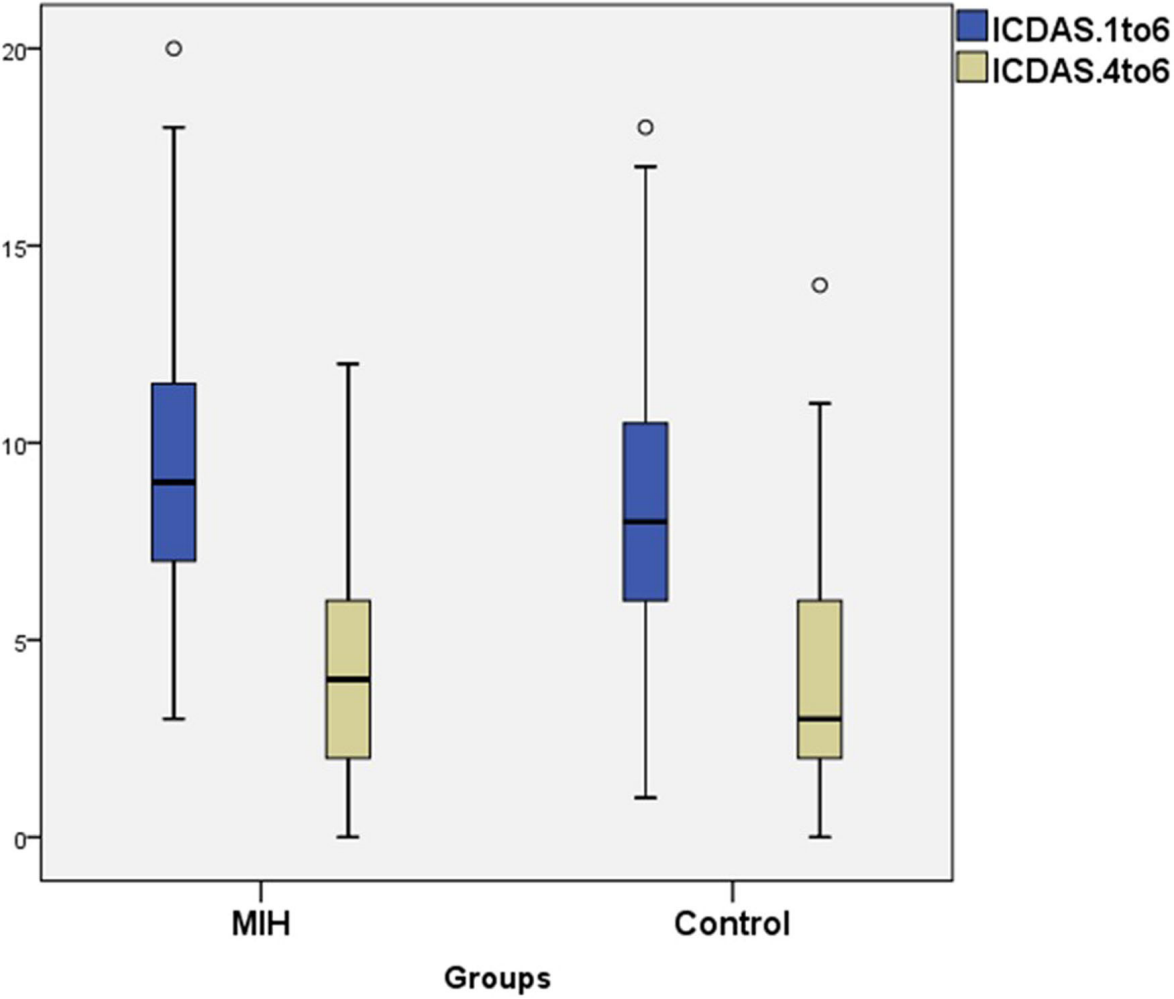
Box plot illustrating the median value of ICDAS scores in both the MIH and the control groups.

ICDAS II (4–6) scores of cavitated lesions recorded mean 4.41 (±2.62, median 4, range 0–12) in the MIH group, in comparison to mean 4.24 (±3.16, median 3, range 0–14) in the control group. This difference was not statistically significant (*p* = 0.469) as shown in Fig. 1.

The analysis for both the DMFT/deft and ICDAS II scoring systems was done at the tooth level.

Kappa coefficient showed a good level of agreement between the examiners of 0.89.

### OHRQoL in MIH and control groups

The mean value for CPQ_8–10_ in the MIH group was 24.67 (±11.84; median 23; range 6–55) in comparison to 21.04 (±12.3; median 18; range 2–54) for the control group, with no significant difference between groups (*p* = 0.109). Regarding the scores of the subdomains, there was no significant difference between groups for oral symptoms (*p* = 0.33), emotional well-being (*p* = 0.236), and social well-being (*p* = 0.676), only for the functional limitations, MIH group recorded mean 6.69 (±3.76; median 7; range 0–16), in comparison to mean 4.43 (±3.83; median 4; range 0–14) in the control group, with a statistically significant difference between groups (*p* = 0.003) as shown in Table 3.

**Table 3:** Comparison of the total score and the four domains of CPQ_8–10_ in MIH and control groups (Mann Whitney *U* test)

CPQ_8–10_ scores		Mean	Std. dev	Median	95% Confidence interval for mean		Min	Max	*P* value
					Lower bound	Upper bound			
Oral symptoms (5)	MIH	10.18	3.56	10.00	9.17	11.18	4.00	18.00	0.336 ns
	Control	9.43	3.73	9.00	8.38	10.48	2.00	17.00	
Functional Limitations (5)	MIH	6.69	3.76	7.00	5.63	7.74	0.00	16.00	0.003*
	Control	4.43	3.83	4.00	3.35	5.51	0.00	14.00	
Emotional well-being (5)	MIH Control	4.80 3.73	5.04 4.29	4.00 2.00	3.39 2.52	6.22 4.93	0.00 0.00	20.00 14.00	0.236 ns
Social well-being (10)	MIH Control	3.00 3.45	3.53 3.97	2.00 3.00	2.01 2.34	3.99 4.57	0.00 0.00	18.00 15.00	0.676 ns
Total CPQ (25)	MIH Control	24.67 21.04	11.84 12.30	23.00 18.00	21.34 17.58	28.00 24.50	6.00 2.00	55.00 54.00	0.109 ns

Significance level *P* ≤ 0.05, *significant, *ns* non-significant

As for emotional well-being only question no.11 “ How often have you had been upset because of your teeth or mouth?”, MIH group recorded mean 1.67 (±1.52; median 2; range 0–4), in comparison to mean 1.04 (±1.34; median 0; range 0–4) in the control group, with a statistically significant difference between groups (*p* = 0.038)

### OHRQoL related to the severity of MIH

**Fig. 2:**
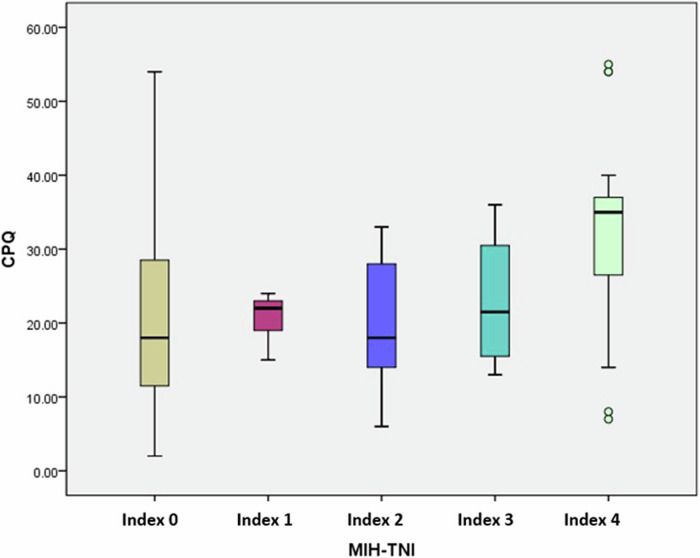
Box plot illustrating the median value of total CPQ scores of the control group(MIH-TNI 0) and the MIH group according to its severity by the MIH-TNI grading index (MIH-TNI 1,2,3,4).

**Fig. 3:**
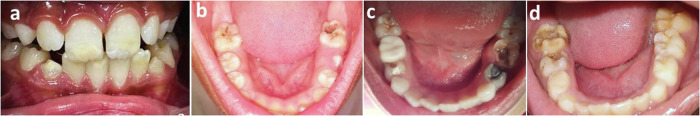
MIH cases classified according to MIH-TNI index. (**a**) Index 1(without hypersensitivity, without defect) in upper central incisors, (**b**) Index 2b (without hypersensitivity, with defect extension ≥1/3 ≤2/3) in lower left first permanent molar, (**c**) Index 3 (with hypersensitivity without defect) in lower right first permanent molar, (**d**) Index 4c (with hypersensitivity, with defect extension ≥2/3) in lower right first permanent molar.

Regarding CPQ_8–10_ scores, a significantly higher value (*p* = 0.011) was recorded in patients with MIH-TNI 4 mean 32.16 (±13.9; median 35; range from 7 to 55) in comparison to MIH-TNI 3 mean 23 (±9.97; median 21; range from 13 to 36), MIH-TNI 2 mean 19.65 (±8.09; median 18; range from 6 to 33) and MIH-TNI 1 mean 20.6 (±3.65; median 22; range from 15 to 24) as shown in Fig. 2. CPQ_8–10_ scores for each domain for MIH-TNI different grades were presented in Table 4. Clinical pictures for MIH cases classified according to MIH-TNI index were shown in Fig. 3.

**Table 4:** Comparison the total score and the four domains of CPQ_8–10_ according to MIH-TNI grades (Mann–Whitney *U* test)

CPQ_8-10_ scores		Groups	Mean	Std. dev	Median	95% Confidence interval for mean		Min	Max	*P* value
						Lower bound	Upper bound			
Oral symptoms		MIH-TNI 1	9.20	1.92	9.00	6.81	11.58	7.00	12.00	0.120 ns
		MIH-TNI 2	9.17	3.65	9.00	7.59	10.75	4.00	18.00	
		MIH-TNI 3	11.00	4.54	12.00	3.76	18.23	5.00	15.00	
		MIH-TNI 4	11.47	3.35	12.00	9.85	13.09	4.00	16.00	
Functional limitations		MIH-TNI 1	6.40	2.07	7.00	3.82	8.97	3.00	8.00	0.383 ns
		MIH-TNI 2	6.04	4.23	5.00	4.21	7.87	0.00	16.00	
		MIH-TNI 3	5.75	4.27	7.00	–1.04	12.54	0.00	9.00	
		MIH-TNI 4	7.73	3.38	8.00	6.10	9.36	0.00	13.00	
Emotional well-being		MIH-TNI 1	2.20	1.48	2.00	0.35	4.04	0.00	4.00	0.058 ns
		MIH-TNI 2	3.21	3.14	3.00	1.85	4.57	0.00	9.00	
		MIH-TNI 3	3.25	2.50	3.50	–0.72	7.22	0.00	6.00	
		MIH-TNI 4	7.73	6.49	8.00	4.60	10.86	0.00	20.00	
Social well-being		MIH-TNI 1	2.80^b^	2.58	2.00	–0.41	6.01	0.00	7.00	0.002*
		MIH-TNI 2	1.21^b^	1.75	0.00	0.45	1.97	0.00	5.00	
		MIH-TNI 3	3.00^b^	3.46	2.00	–2.51	8.51	0.00	8.00	
		MIH-TNI 4	5.21^a^	4.26	5.00	3.15	7.26	0.00	18.00	
CPQ		MIH-TNI 1 (*n* = 7)	20.60^b^	3.64	22.00	16.07	25.12	15.00	24.00	0.011*
		MIH-TNI 2 (*n* = 21)	19.65^b^	8.09	18.00	16.15	23.15	6.00	33.00	
		MIH-TNI 3 (*n* = 7)	23.00^b^	9.96	21.50	7.14	38.85	13.00	36.00	
		MIH-TNI 4 (*n* = 17)	32.15^a^	13.90	35.00	25.45	38.85	7.00	55.00	

Significance level *p* ≤ 0.05, *significant, *ns* non-significant

Within the same comparison, values with the same superscript letter are not significantly different

### OHRQoL between male and female patients

For the MIH group, CPQ_8–10_ scores showed no significant difference between genders (*p* = 0.992), only in Question no. 13 ”How often have you had been shy because of your teeth or mouth?” a significantly higher value (*p* = 0.044) was recorded in females (mean 1.22 ± 1.48, median 0, range 0–4), in comparison to males (mean 0.42 ± 1.02, median 0, range 0–3) and in question no 15” How often have you had worried that you are not as good-looking as others because of your teeth or mouth?”, a significantly higher value (*p* = 0.019) was recorded in females (mean 0.75 ± 1.37, median 0, range 0–4), in comparison to males who recorded score 0 in all cases.

Also, for the control group, CPQ_8–10_ scores showed no significant difference between genders (*p* = 0.148).

### OHRQoL between different age groups

CPQ_8–10_ scores at 8 years showed a statistically significantly (*p* = 0.039) greater value (median = 34.5) in MIH group, in comparison to control group (median = 18), while no statistically significant difference was recorded between MIH and control group at 9 years (*p* = 0.808) and 10 years (*p* = 0.077).

## Discussion

The concern for MIH is increasing worldwide among clinicians and also among patients in relation to their impact on oral health [38]. The main objective of the current study was to study the effect of MIH on OHRQoL in children at the mixed dentition stage and correlate it with the caries experience of these children. The CPQ_8–10_ with its validated Arabic version was used in this study as a widely used instrument to evaluate the OHRQoL [35]. The mean age in the MIH group was 8.94 ± 1.45 years in comparison to 8.8 ± 1.55 years in the control group, which is consistent with the EAPD guidelines, given that first permanent molars and permanent incisors usually erupt at that age and the risk of enamel defects concealment by carious cavities or restorations is restricted [39]. In addition, according to a systematic review published in 2021, the most common age of children examined in MIH studies was 8–10 years [40]. The understanding of the connection between MIH and caries and the overlap between the two conditions represents a clinical challenge and reflects the complexity of diagnosing MIH lesions and carious lesions [41].

Many investigations assessing dental caries in individuals with MIH commonly employ the DMFT index, as suggested by the WHO [20, 42–44]. Although, many studies opt for the DMFT index for assessing caries, yet it has limitations, including its failure to consider the stage of the carious lesion [36, 43, 45]. The ICDAS II index is effective in identifying caries at various stages, encompassing both cavitated and non-cavitated lesions. Furthermore, ICDAS II streamlines the diagnostic process, enhancing its accuracy and standardization in detecting caries [46]. Hence, the ICDAS II system for caries diagnosis with its detailed description was also used in this study as it allows for a more accurate picture of MIH and dental caries relationship [47].

The results of caries screening using the DMFT index showed a significantly higher difference for the MIH group compared to the control group regarding the mean DMF scores for permanent teeth, where both D and F scores were significantly higher in the MIH group. This can be explained by the fact that the hypomineralized enamel surface exhibits higher porosity compared to intact enamel, which facilitates increased biofilm buildup, consequently promoting demineralization [45].

Moreover, concerning that the differential diagnosis between dental caries and post-eruptive enamel breakdowns is quiet challenging, it was previously reported that the scores of DMFT caries index for MIH patients will probably be overestimated where score D can be given for post-eruptive enamel breakdowns not only dental caries and score Fand M is given for restorations or extractions treating dental caries, post-eruptive enamel breakdowns or combinations of both [43].

A systematic review published in 2017 concluded that the DMF index was higher in children with MIH than in children without MIH, but it is worth noting that none of the studies included were classified as high-quality studies [38].

Combining both DMFT index for permanent teeth and deft for primary teeth showed a mean score of 4.76 (±2.84) in the MIH group, in comparison to mean 4.51 (± 3.12) in the control group with a non-statistically significant difference. Similarly, a previous study recorded dmft/DMFT mean score of 5.04 (±3.73) for the MIH group in comparison to 5.49 (±3.84) for the control one showing a comparable caries experience [42].

In this study, the ICDAS II index was employed, which takes into account both the stages of the lesion and the child’s caries history [46].

The ICDAS II (1–6) scores including the non-cavitated lesions showed no difference between MIH patients and control ones as the early signs of dental caries as white spot lesions usually occur in areas of plaque stagnation in the cervical areas of smooth surfaces where enamel hypomineralization rarely occurs [48]. So, no overestimation of the ICDAS scores of 1 and 2 in MIH patients was presented.

Regarding the ICDAS II (4–6) scores of cavitated lesions with dentinal involvement in both primary and permanent teeth collectively, there were no statistically significance difference between the MIH and control groups. To our knowledge, there was only one study conducted before for comparisons of the ICDAS II scores in our same age group, their results showed that caries is far greater in surfaces with severe MIH than in surfaces with mild MIH or no MIH, as they explained that creamy and brownish opacities are more porous and susceptible to post-eruptive enamel breakdowns that in return worsen caries, however, there were two differences than our study where there was subgrouping of MIH to two forms, mild and severe one and MIH and caries was assessed by two different examiners [49]. Another study compared caries experience in children aged 3–5 years with and without hypomineralized second primary molars, there was no significant difference in ICDAS II codes (2–6) or (4–6) at their tooth surface level or in the overall caries experience [50].

The comparable ICDAS II (4–6) scores in the two groups in our study which represents the more severe form of caries involvement may reflect their similar impact on oral symptoms.

Regarding the effect of MIH on the OHRQoL, the impact of MIH only on the domain of functional limitations was manifested. In a systematic review published in 2021 [40], only two articles showed a significant impact of MIH on all domains of OHRQoL, Gutiérrez et al. [51] in 2019 and Velandia et al. [2] in 2018.

On the other hand, in agreement with our results, other studies evaluated the effect of MIH on OHRQoL in children and concluded that the presence of MIH had no significant impact on the OHRQoL according to children’s self-reported perceptions, these studies was conducted in developing countries similar to our study [24, 52].

Demographic and socioeconomic factors, along with the organization of children’s dental services, vary significantly worldwide which may explain these differences between studies. This controversy in the results of previous studies also prompts inquiry into potential cultural variances in esthetic perception and the psychological impact of esthetics on self-well-being [53].

Regarding the functional limitations caused by MIH, previous studies explained this impact as a result of hypersensitivity, which is the main issue that appeared to stem from challenges with eating and maintaining oral hygiene [54].

Meanwhile, when considering the clinical severity of MIH, significantly higher CPQ_8–10_ scores were recorded in patients with MIH-TNI 4 in comparison to other categories of MIH and to the control group. Patients with MIH-TNI 4 show the two most important clinical symptoms of MIH which is hypersensitivity and disintegration. Joshi et al. study showed increased CPQ_8–10_ scores with increasing severity reflecting a more impaired OHRQoL, their findings demonstrated statistically significant variances between the control group and the MIH-TNI groups of index 2, 3, and 4, but no statistically significant difference was recorded between the control group and MIH-TNI 1 [42]. Other studies also showed that the more severe the MIH, the greater the impact on OHRQoL where hypersensitivity, enamel fractures, atypical cavities adversely affected the OHRQoL [20, 41].

CPQ_8–10_ scores for male and female patients showed no significant difference between them in the MIH group, however only two questions concerning dental esthetics and their social influence showed a significantly higher value in females compared to males. It is reported that females are more concerned with dental esthetics and smile satisfaction than males [55]. Interestingly, among facial esthetics, the smile appears to be critical for adolescents as well as for children younger than 10 years of age and influence their social perception [56]. Concerning the OHRQoL between different age groups, CPQ_8–10_ scores only at 8 years showed a statistically significantly greater value in the MIH group, in comparison to the control group, this could be attributed to hypersensitivity where a previous study conducted on MIH patients between 6 and 18 years of age with a mean of 10.9 (±2.9) years showed that the degree of hypersensitivity is significantly higher in individuals aged ≤8 years [15].

Among the limitations of the current study, the collected data represent the individual’s perception at the time of assessment. Therefore, a longitudinal study design is necessary to examine the influence of MIH on OHRQoL over time with respect to different socioeconomic and cultural differences.

## Conclusion

Children with and without MIH showing similar overall caries experience recorded by ICDAS II showed no difference in their OHRQoL evaluation except for functional limitations. However, the presence of the severe form of MIH including hypersensitivity and enamel disintegration negatively impacted those children’s OHRQoL.

## Data Availability

The datasets used and/or analyzed during the current study are available from the corresponding author on reasonable request.
